# Retraction Note: hPMSC transplantation restoring ovarian function in premature ovarian failure mice is associated with change of Th17/Tc17 and Th17/Treg cell ratios through the PI3K/Akt signal pathway

**DOI:** 10.1186/s13287-022-03173-8

**Published:** 2022-09-14

**Authors:** Na Yin, Yanlin Wang, Xueyan Lu, Ranran Liu, Lianshuang Zhang, Wei Zhao, Wendan Yuan, Qianqian Luo, Hao Wu, Xiying Luan, Hongqin Zhang

**Affiliations:** 1 Department of Histology and Embryology, Binzhou Medical University, 346 Guanhai Rd, Yantai, Shandong China; 2 Reproductive Medicine Center of the Affiliated Hospital of Binzhou Medical College, Binzhou, Shandong China; 3 Reproductive Medicine Center of the Affiliated Hospital of Binzhou Medical College, Yantai, Shandong China; 4 Research Institution of Reproductive Medicine, Binzhou Medical University, Yantai, Shandong China; 5 Basic Medicine College, Binzhou Medical University, Yantai, Shandong China; 6 Department of Morphology Laboratory, Binzhou Medical University, Yantai, Shandong China; 7 Clinical Medical School, Binzhou Medical University, Yantai, Shandong China; 8 Department of Immunology, Binzhou Medical University, 346 Guanhai Rd, Yantai, Shandong China

## Retraction Note: *Stem Cell Research & Therapy* (2018) 9:37 10.1186/s13287-018-0772-x

The Editor-in-Chief has retracted this article at the Corresponding Author's request. After publication, concerns were raised regarding partial image overlap between figures in this article and another article by the same authors submitted and published within a close time frame [1]. Specifically:

- Fig. 1A appears to present the same data as Fig. 2A in [1];
- Fig. 1B and D appear to overlap with Fig. 2B and D in [1], respectively;
- Fig. 3A appears to originate from a different section in the same sample as Fig. 5A in [1];
- Fig. 4A-1 and A-3 appear highly similar to Fig. 6C and A in [1], respectively;
- Fig. 5A Akt lane 1 and GAPDH lanes 1–3 appear highly similar to Fig. 7A FSHR lane 1 and GAPDH lanes 1–3 in [1], respectively

The Authors have stated that the data in Figs. 1–4 correspond to the experiments performed in this article, and that the conclusions of this article are not affected by these errors. However, they are unable to explain the similarities in the western blot images. The authors have therefore requested a retraction due to loss of confidence in the presented data.

All authors agree to this retraction.
